# Supplementary material for: From sequence to enzyme mechanism using multi-label machine learning
Source: BMC Bioinformatics. 2014 May 19;15:150. doi: 10.1186/1471-2105-15-150 (PMC4229970; doi:10.1186/1471-2105-15-150)
Supplement: Additional file 2 — Java code of ml2db. Additional file ml2db_code.tar.gz contains the Java source code to run the multi-label machine learning experiments and save the results to database. The code’s Javadoc is included. [file 1471-2105-15-150-S2.zip › additional file 2/ml2db/ml2db/doc/main/ml2db/al/utility/globaldistance/class-use/GlobalDistanceMetric.html]

Uses of Class main.ml2db.al.utility.globaldistance.GlobalDistanceMetric


JavaScript is disabled on your browser.


- Overview
- Package
- Class
- Use
- Tree
- Deprecated
- Index
- Help

- Prev
- Next

- Frames
- No Frames

- All Classes

## Uses of Class main.ml2db.al.utility.globaldistance.GlobalDistanceMetric

- Packages that use GlobalDistanceMetric

  | Package | Description |
  | --- | --- |
  |  |  |
  | --- | --- |
  | main.ml2db.al.utility.globaldistance |  |
  | main.ml2db.al.utility.hybrid |  |
  | test.ml2db.al.utility.globaldistance |  |
- - ### Uses of GlobalDistanceMetric in main.ml2db.al.utility.globaldistance

    Methods in main.ml2db.al.utility.globaldistance that return GlobalDistanceMetric

    | Modifier and Type | Method and Description |
    | --- | --- |
    |  |  |
    | --- | --- |
    | `GlobalDistanceMetric` | GlobalPositionStore.`getPositioningMetric()` |

    Constructors in main.ml2db.al.utility.globaldistance with parameters of type GlobalDistanceMetric

    Constructor and Description

    `GlobalPositionStore(int hashInitialSize,
    GlobalDistanceMetric positioningMetric)`
  - ### Uses of GlobalDistanceMetric in main.ml2db.al.utility.hybrid

    Subclasses of GlobalDistanceMetric in main.ml2db.al.utility.hybrid

    | Modifier and Type | Class and Description |
    | --- | --- |
    |  |  |
    | --- | --- |
    | `class` | `GlobalDistanceWithConfidenceMetric` Combines a Positioning metric (a metric that considers positively if an instance is close to other unlabelled instances and distant from other labelled instances) with the average certainty of the instance prediction (that is, how distant the average of confidence over all labels is from 50%) |
  - ### Uses of GlobalDistanceMetric in test.ml2db.al.utility.globaldistance

    Methods in test.ml2db.al.utility.globaldistance with parameters of type GlobalDistanceMetric

    | Modifier and Type | Method and Description |
    | --- | --- |
    |  |  |
    | --- | --- |
    | `void` | GlobalDistanceMetricTest.`printInst(GlobalDistanceMetric position, IdInstance i)` |

- Overview
- Package
- Class
- Use
- Tree
- Deprecated
- Index
- Help

- Prev
- Next

- Frames
- No Frames

- All Classes
